# Nimodipine-Loaded Pluronic^®^ Block Copolymer Micelles: Preparation, Characterization, *In-vitro* and *In-vivo* Studies

**Published:** 2016

**Authors:** Farzaneh Sotoudegan, Mohsen Amini, Mehrdad Faizi, Reza Aboofazeli

**Affiliations:** a *Department of Pharmaceutics, School of Pharmacy and Protein Technology Research Center, Shahid Beheshti University of Medical Sciences, Tehran, Iran. *; b *Department of Pharmaceutical Chemistry, Faculty of Pharmacy, Tehran University of Medical Sciences, Tehran, Iran. *; c *Department of Pharmacology and Toxicology, School of Pharmacy, Shahid Beheshti University of Medical Sciences, Tehran, Iran.*

**Keywords:** Nimodipine, Blood brain barrier, Polymeric micelles, Pluronics, FITC

## Abstract

Nimodipine (NM), as a lipophilic calcium channel blocker indicated for the prevention and treatment of neurological disorders, suffers from an extensive first pass metabolism, resulting in low oral bioavailability. Polymeric micelles, self-assembled from amphiphilic polymers, have a core-shell structure which makes them unique nano-carriers with excellent performance as drug delivery. This investigation was aimed to develop NM-loaded polymeric micelles and evaluate their potential to cross the blood brain barrier (BBB). Micelles from Pluronics^®^P85, F127 and F68 were fabricated for the delivery of NM, using thin film hydration and direct dissolution techniques. Critical micelle concentration of the drug-free micelles was determined by pyrene ﬂuorescence spectroscopy. Dynamic light scattering showed that in most cases, micelles less than 100 nm and low polydispersity indices were successfully developed. Transmission electron microscopy demonstrated spherical shape of micelles. The NM-loaded micelles were also characterized for particle size, morphology, entrapment efficiency, drug loading , in vitro drug release in phosphate buffer and artificial cerebrospinal fluid (CSF). Stability was assessed from size analysis, clarity of dispersion on standing and EE(%), following 3 months storage at room temperature. The *in-vitro* release of NM from polymeric micelles presented the sustained-release profile. Animal studies revealed the existence of fluorescein 5-isothiocyanate-labeled micelles in rat CSF following intraperitoneal administration, proving that the micelles crossed the BBB. Anticonvulsant effect of NM was shown to be significantly greater than that of NM solution. Our results confirmed that Pluronic micelles might serve as a potential nanocarrier to improve the activity of NM in brain.

## Introduction

In spite of significant improvements in research in the area of brain and central nervous system (CNS) disorders, CNS diseases are still the world›s principal cause of disabilities. Various physical barriers between blood and the CNS, including the blood-brain barrier (BBB), protect the CNS against potentially harmful endogenous and exogenous substances (1-4). A network of brain tissue capillaries which form a structural and chemical barrier between the brain and systemic circulation, are composed of specialized endothelial cells that lack pores to allow rapid exchange of molecules and are connected by extensive tight junctions that form a high-resistance barrier and severely restrict cell permeability. Under normal physiological conditions, this low BBB permeability prevents transport of bacteria and restricts movement of 100% of large molecules and 98% of small molecules from the systemic circulation to the brain (5, 6). In order to enter the brain, molecules need to be less than 400 Daltons and hydrophobic. Too large or too hydrophilic molecules can pass BBB as substrates of active efflux transporters which are important determinants of drug distribution to, and elimination from, the CNS (7).

Neuropharmaceutical research is associated with a significant challenge which is the requirement of neuropharmaceuticals to cross blood-CNS barriers, since they present a major obstacle for effective delivery of potential CNS medicines (4, 8). A few strategies have been proposed to disrupt the BBB for drug delivery, including trans-cranial approach using neurosurgery-based intrathecal catheters, bypassing the BBB via the olfactory route, shrinkage of the endothelial cells and opening of the tight junctions using various compounds (e.g., mannitol, ethanol or dimethylsulfoxide, alkylating agents, etc.) (6, 9). However, several major disadvantages are associated with each of these strategies. In parallel, for those drug candidates that are not capable of successfully crossing the blood-CNS barriers, there is the possibility of transvascular delivery to the brain. It has been demonstrated that the efflux transporter P-glycoprotein (P-gp) can actively transport lipophilic drugs out of the capillary endothelial cells in brain, leading to minimizing or avoiding neurotoxic adverse effects of drugs. However, efflux transporters may also limit the central distribution of drugs that are beneficial to treat CNS diseases. Therefore, modulation of efflux transporters at the BBB can be considered as a novel strategy to enhance the penetration of drugs into the brain, especially for drug-resistant CNS diseases (10, 11).

An alternative way for drug delivery to the brain is to use delivery systems that facilitate the movement of drug candidate across the blood-CNS barrier (8, 9, 12). Polymeric micelles are one of the promising nanomedicine-based technologies which have been evaluated as nano-carriers for anticancer drugs (13-18). Polymeric micelles are kinetically stable nanoparticles with a core-shell structure which are made from amphiphilic block copolymers with hydrophilic and hydrophobic chains that self-assemble in water above the critical micelle concentration. The hydrophobic core of these nano-carriers could serve as a microenvironment for solubilizing poorly water soluble drugs through hydrophobic interaction and/or hydrogen bonding and protect the enclosed compounds from inactivation in biological media, while exposing their hydrophilic shells to the external environment (9, 13). In addition, these systems exhibit many advantages, among which small particle size (less than 100 nm), targeting ability, ease of preparation and prolonged circulation time (the ability to escape from renal exclusion and reticuloendothelial system) could be mentioned (9, 13, 19-24). Pluronic block copolymers (PBC), named also as poloxamers, are amphiphilic synthetic polymers containing hydrophilic poly(ethylene oxide) (PEO) blocks and hydrophobic poly(propylene oxide) (PPO) blocks which are arranged in tri-block (PEO-PPO-PEO) structures. These polymers not only serve as inert carriers, but also were shown to function as biological response modifiers and inhibit P-gp that sentisize multidrug resistance tumor cells to various anticancer agents (13, 18, 25-29).

Nimodipine (NM) is a dihydropyridine calcium channel blocker which has been shown to selectively regulate calcium channels, dilate cerebral arterioles and thereby increase cerebral blood flow (30). This drug is indicated for the treatment of a range of cerebrovascular disorders, focused mainly in the prevention and treatment of delayed ischemic neurological deficits in patients with subarachnoid hemorrhages as a result of sustained cerebral vasospasm. This drug is also indicated in other cerebrovascular disorders, such as ischemic stroke, multi-infarct dementia and hypotension-induced memory impairment (31). However, its clinical performance is limited because it is subjected to extensive first-pass metabolism in liver which leads to very low oral bioavailability and hence only a small fraction of the administered dose is delivered to the brain. Also, NM is highly lipophilic which necessitates its administration as a parenteral formulation containing organic solvents, such as ethanol. NIM ethanolic injections may cause local adverse reactions (including pain, inflammation, phlebitis) at the administration site (31-35). Therefore, for safety considerations and improvement of therapeutic effects, new NM delivery systems are needed to be developed in an attempt to facilitate its targeting to the brain.

The present investigation was planned to develop and characterize NM-loaded, Pluronic-based nano-carriers. Since PBCs were shown to inhibit P-gp (29, 36), it was hypothesized that these polymeric micelles may be used to transport NM across the BBB and enhance its brain delivery for treatment of CNS disorders. Single micelles were prepared with various PBCs (namely, F68, P85 and F127) and characterized in terms of critical micelle concentration, particle size distribution and morphological observation. To investigate their feasibility as carriers for drugs, NM-loaded polymeric micelles were produced by thin-film hydration and direct dissolution methods and evaluated for size distribution, drug loading, encapsulation efficiency, drug precipitation, stability, *in-vitro* release in phosphate butter and cerebrospinal fluid (CSF) media. Fluorescein isothiocyanate (FITC) was loaded into the P85-based micelles for the detection of micelles in CSF. Finally, animal studies were carried out to examine the ability of the NM-loaded polymeric micelles for passing through the BBB and evaluate their anticonvulsant activity.

## Experimental

*Materials*

Hydrophilic Pluronic^®^ block copolymers (PBC), including P85, F127, and F68, were commercially available from BASF Corporation (New Jersey, USA) and used without additional purification. The structure, average molecular weight and HLB values of the copolymers used in this investigation are shown in Table 1. Nimodipine, pyrene, pentylenetetrazole (PTZ), fluorescein isothiocyanate (FITC), N,N›-dicyclohexylcarbodiimide (DCC), N-hydroxysulfosuccinimide (NHS), potassium dichromate, sodium chloride, potassium chloride, magnesium chloride, calcium chloride, HEPES, ethylenediamine and glucose were purchased from Sigma-Aldrich Chemical Company (Missouri, USA). Polysorbate 80 (Tween^®^ 80) was provided from Fluka Chemicals Company (Seelze, Germany). 1-ethyl-3-[3-dimethylaminopropyl] carbodiimide hydrochloride (EDC) was supplied from Pierce (Texas, USA). All other chemicals and solvents were obtained from Merck Company (Darmstadt, Germany).

*Preparation of drug-free polymeric micelle solutions*

To prepare polymeric micelles, appropriate amounts of PBCs were added to 10 mL deionized water (DI, with partial conductivity of 0.054 µs/cm) and the mixtures were stirred for 6 h at 100 rpm, at room temperature. To evaluate the micelle-forming ability of PBCs, polymeric solutions were prepared with each individual PBC at two different concentrations (Table 2).

*Determination of Critical Micelle Concentration (CMC)*

CMC is a concentration above which an amphiphilic copolymer could form core-shell structured micelles. The CMC values of PBCs in deionized water were estimated by using a standard pyrene fluorescence procedure. Pyrene, as a widely used fluorescence probe, is commonly used for the study of micelle formation and determination of CMC (9, 25, 37-40). Pyrene solution of acetone was transferred into glass bottles and the solvent was allowed to evaporate under mild heating. Polymer solutions with different concentrations were then added to the glass bottles. The final pyrene concentration was kept constant at 6 × 10^-7^ M. The solutions were allowed to stand for 24 h at room temperature to achieve equilibrium. Fluorescence spectra were recorded by a spectrofluorometer (Hitachi, Tokyo, Japan) at room temperature. Emission wavelength was set at 390 nm and the micellization of Pluronic block copolymers was characterized by employing the excitation intensity ratio of I_339_ / I_333_ (41). Upon formation of micelles, pyrene would move into the inside of the micelles from the aqueous phase, resulting in an alteration in the intensity ratio. The I_339_ / I_333_ value was then plotted vs. the logarithm of polymer concentration. The CMC value was taken from the intersection of a fitted line of the curve at low polymer concentration (flat region) with a fitted line on a rapidly rising part of the curve. The CMC was assumed where a steep increase in fluorescence intensity was observed (42, 43).

*Morphological examination*

To confirm the formation of polymeric micelles with PBCs, a sample of micellar solution was dropped on a TEM copper grid and negatively stained by the addition of one drop of phosphotungstic acid solution (2%, w/v). The grid was finally examined under a transmission electron microscope (TEM, Zeiss, EM10C, Oberkochen, Germany), with the accelerating voltage of 80 kV.

*Determination of particle size*

The mean particle size, size distribution and polydispersity index (PDI) of the polymeric micelles were determined at 25 °C by dynamic light scattering (DLS), using Malvern Zetasizer (Nano-ZS; Malvern Instruments, Worcestershire, UK), equipped with a Nano ZS^®^ Software for data acquisition and analysis. The analysis was performed three times to determine the mean values. Each analysis was performed at the angle detection of 175 ° and lasted for 60 s. 

*Preparation of NM-loaded polymeric micelles, by thin-film hydration method*

Briefly, 2 mg NM and an appropriate amount of the Pluronic polymers (Table 2) were accurately weighed and dissolved in 10 mL acetonitrile or methanol in a round-bottom flask. The solvent was slowly evaporated at 60 °C under reduced pressure using a rotary evaporator (Heidolph, Schwabach, Germany), revolving at 120 rpm such that a thin dry film of NM-copolymer matrix was formed on the inner wall of the flask. The residual amount of the solvent remaining in the film was removed under vacuum overnight at room temperature. The dried film was hydrated with deionized water (10 mL), while the hydration temperature was kept constant at 60 °C. The mixture was stirred at 120 rpm for one hour in rotary evaporator to obtain a polymeric micelle solution (21). The unincorporated drug and polymer aggregates were removed by the following techniques:

a) *Membrane filter*: Formulations were filtered through 0.45 µM filter membrane (NYLON Membrane Filter, Vertical). Sediments on the filter were then dissolved in methanol in order to determine the percent of precipitated NM. 

b) *Centrifugation*: NM-loaded micelles were centrifuged (Beckman, Fullerton, Canada) at 12,000 rpm for 5 min at room temperature. Sediments at the bottom of the centrifuge tubes were then dissolved in methanol in order to determine the percent of precipitated NM. 

c) *Filtration-Centrifugation*: Micellar solutions were filter-centrifuged using Vivaspin^®^ centrifugal concentrator tubes, at 12,000 rpm for 15 min at room temperature. Sediments on the filter were then dissolved in methanol in order to determine the percent of precipitated NM. 

Particle size, PDI, entrapment efficiency (EE) and drug loading (DL) percentages were then measured in the filtered, centrifuged and filter-centrifuged polymeric micelle solutions.

*Preparation of NM-loaded polymeric micelles, by direct dissolution method*

*Method 1*: An appropriate amount of the Pluronic polymers (Table 2) was added to 10 mL DI and the solution was stirred for 3 h at 100 rpm. Once clear solution was obtained (approximately 3 h for polymers to be dissolved thoroughly in water), 2 mg NM was added and the mixture was stirred at 100 rpm for 3, 12 and 24 h at room temperature (25 °C). 

*Method 2*: Alternatively, 2 mg of the drug and an appropriate amount of the polymer were added simultaneously to DI and the solutions were stirred at 100 rpm for 3, 12 and 24 h at room temperature (25 °C).

For both methods, the precipitated NM was separated from polymeric micelle solutions by filtering through 0.45 µM filter membranes (NYLON Membrane Filter, Vertical). The sediments on the filter were dissolved in methanol and assayed, and the filtered solutions were used for the determination of particle size, PDI, EE and DL.

*Determination of entrapment efficiency (EE), drug loading (DL) and precipitated drug percentages*

NM shows a strong absorption band at the wavelength of 236 nm whereas methanol and the PBCs used in the investigation showed no absorbance at this wavelength. Therefore, a UV spectrophotometric method (Shimadzu, model UV 240, Tokyo, Japan) was employed to determine (in triplicate) the EE, DL and precipitated drug percentages, using the following equations:

$\mathrm{DL}(\%)=\frac{\mathrm{weight of the drug in micelles}}{\mathrm{weight of the feeding polymer and drug}}\times 100$

$\mathrm{EE}(\%)=\frac{\mathrm{weight of the drug in micelles}}{\mathrm{weight of the feeding drug}}\times 100$

Precipitated drug (%)=original drug added -drug remained in the supernatant original drug added×100

*In-vitro release study of NM from micelles in phosphate buffer solution (PBS)*

The release profile of NM from developed polymeric micelles was evaluated using the dialysis bag method. Dialysis bags (cut-off of 35,000 g/mole, Spectera/Por 7, Specteram Laboratories, Shanghai, China) were soaked in distilled water for 24 h and kept under refrigeration until use. Ten milliliters of NM-loaded polymeric micelle solutions (containing the equivalent to 2 mg of NM) were placed in dialysis membranes. The bags were then sealed with clips at both ends and submerged into 200 mL of PBS medium (0.2 M, pH 7.4, sink condition) containing 0.1% (w/v) of Tween^®^ 80. Tween^®^ 80 is a low molecular weight, non-ionic surfactant that can be added to the release medium in order to maintain perfect sink conditions for hydrophobic drugs (13, 22). The entire assembly was kept at 37 °C with continuous magnetic stirring at 100 rpm for 72 h. The aliquots of 4 mL were then withdrawn from the dissolution medium at predetermined time intervals for 72 h (0, 15, 30 min and 1, 2, 4, 6, 8, 10, 12, 24, 36, 48, 60, 72 h) and replaced with an equivalent volume of fresh medium to maintain the volume of the medium at 100 mL and ensure creation of a perfect sink condition. Due to the presence of Tween^®^ 80 which shows some absorbance at 236 nm, λ_max_ of 354 nm of NM was used to measure the drug release. At this wavelength, the absorbance of PBS, Tween^®^ 80 and polymers could be negligible. Samples were assayed by UV spectrophotometer in triplicate and the amount of the drug released in the medium was calculated on the basis of a calibration curve, using different concentrations of free drug in PBS. As a control solution, a hydro-alcoholic solution of NM at the concentration of 0.2 mg/mL (23.7%v/v of ethanol) was used. Care was taken during the experiments to protect NM against light.

*In-vitro release study of NM from micelles in artificial cerebrospinal fluid (CSF)*

To prepare artificial CSF (osmolarity of 320 mOsm, at pH 7.4), 150 mM of NaCl, 3.5 mM of KCl, 2 mM of MgCl_2_, 1.2 mM of CaCl_2_, 10 mM of HEPES and 20 mM of glucose were added to 200 mL of distilled water. The solution was stirred for 2 h at 100 rpm at room temperature (44). Sealed dialysis bags, filled with 10 mL of the drug-loaded polymeric micelles (containing equivalent to 2 mg of NM) were submerged into 200 mL of the artificial CSF solution at 37 °C with continuous stirring speed at 100 rpm for 72 h. 

The CSF solutions containing released NM were collected at predetermined time points for 72 h (as before) and replaced with an equivalent volume of fresh CSF medium to maintain the volume of the medium at 100 mL and ensure the sink condition. The maximum wavelength (λ_max_) of 354 nm of NM was used to measure the drug release, since at this wavelength, the absorbance of the CSF components and the polymers could be negligible. Samples were assayed by UV spectrophotometer in triplicate and the amount of the drug released in the artificial CSF was calculated, using a calibration curve constructed with serially diluted concentrations of NM in CSF. As a control sample, ethanolic solution of NM at the concentration of 0.2 mg/mL (23.7% v/v of ethanol) was used.

*Determination of polymeric micelles stability*

Blank and NM-loaded polymeric micelle solutions were prepared by direct dissolution method (method 2) and stored in glass vials at 25 °C for 3 months and the samples were monitored after 1, 2 and 3 months for any time-dependent changes in particle size, clarity of dispersion on standing and EE after filtration of precipitated drug. No special precautions were taken in order to improve the stability of the micelles. In case of instability (liquid-phase separation and high turbidity due to the formation of large aggregates with a wide size distribution), samples were introduced to sonication for 2 and 30 min, in an attempt to evaluate the reversibility polymeric micelle formation.

*Synthesis of FITC-labeled Pluronic P85 (P85-FITC)*

In order to load FITC into polymeric micelles for visualization of micelles in CSF, EO_26_–PO_40_–EO_26_ (2 g) was dissolved in 100 mL of potassium dichromate solution (5% w/v). Concentrated sulfuric acid (2 mL) was added drop-wise to this solution, kept in an ice bath and the mixture was stirred for 24 h at room temperature. Ethyl acetate was used for the separating of the organic phase. The solvent in the polymeric organic phase was evaporated by the rotary evaporator (Heidolph, Schwabach Germany). The NMR (Bruker, Rheinstetten, Germany) and FTIR spectra (Nicolet 550, Minnesota, USA) were studied to monitor the change of hydroxyl to carboxylic acid group.

**Scheme 1:**
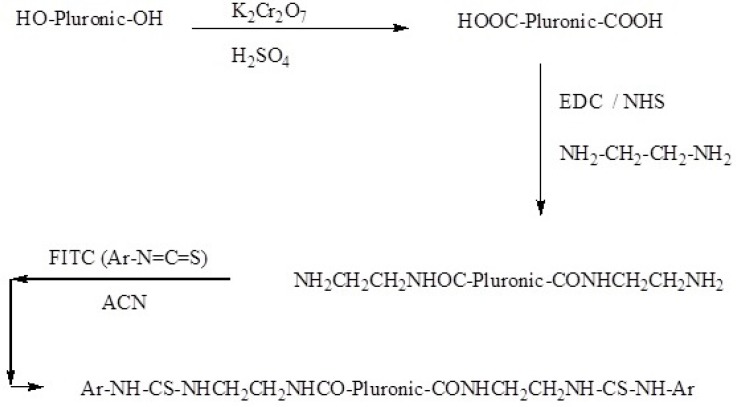
Synthesis of FITC-labeled Pluronic P85 (P85-FITC).

In the next step, FITC-labeled Pluronic copolymer was prepared. DCC (4 g) and NHS (2 g) were added to a solution of the polymer (2 g) in 1 L of chloroform at room temperature with stirring. Ethylenediamine (20 mL) was then added to the solution and the mixture was stirred at 100 rpm for 24 h. The completion of the reaction was examined by FTIR spectroscopy. FITC (200 mg) and triethylamine (4 mL) were added to a solution of polymer-COOH (2 g) in acetonitrile and the mixture was subsequently stirred for 24 h at room temperature. NMR spectroscopy was employed to confirm the FITC conjugation to the polymer (Scheme 1).

*Evaluation of P85 Pluronic micelle penetration into blood brain barrier*

A rat model was employed to evaluate the ability of the polymeric micelles for passing through the BBB. Adult male Wistar rats weighting between 200-250 g were supplied by Experimental Animal Laboratory of School of Pharmacy, Shahid Beheshti University of Medical Sciences (Tehran, Iran). Animals were provided with a standard laboratory diet and all experiments were carried out in accordance with guidelines evaluated and approved by the Ethics Committee of Shahid Beheshti University of Medical Sciences (Tehran, Iran). FITC-labeled P85 micelles were injected intraperitoneally (IP) in 3 animals and after 40 min, their spinal fluids were aspirated (45) and evaluated for the potency of the fluorescence color, using a fluorescence microscope (Hitachi, Tokyo, Japan).Furthermore, both NM-loaded micellar solution and NM ethanolic solutions (20 mg NM in 100 mL of 20% ethanol/water mixture) were also prepared separately and injected IP to 10 laboratory rats and their spinal fluids were aspirated 40 and 30 min, respectively. The samples were analyzed by a previously described HPLC method (46).

*Evaluation of anticonvulsant activity*

In pharmacological evaluation, the anticonvulsant effect of NM, both as a free drug and loaded into the polymeric micelles, was determined by PTZ-induced lethal dose convulsion (47). Male NMRI mice, supplied by Experimental Animal Laboratory of School of Pharmacy, Shahid Beheshti University of Medical Sciences (Tehran, Iran), weighing in the range of 17–20 g were used in this experiment. The animals were housed in a temperature-controlled condition and 12 h light/dark cycle. All pharmacological experiments were performed between 9:00 and 15:00. Standard mouse diet and water were freely available except during the experiment. Thirty minutes before the experiment, the animals were selected randomly and transferred into individual cages and allowed to acclimatize before injections. Freshly prepared NM-free ethanolic solution and NM ethanolic solution (20 mg NM in 100 mL of 20% ethanol/water mixture) were injected IP to ten male NMRI sterile mice, 30 min before injection of PTZ (100 mg/Kg). NM-loaded P85 micellar solution (F4) and blank P85 micellar solution (F4) were also freshly prepared and administered IP to ten mice, 40 min before injection of PTZ (100 mg/Kg). PTZ aqueous solution was then injected and the mice were observed carefully for further 30 min and the dead mice were counted. 

The protocol for all animal experiments was approved by the Institutional Animal Care and Use Committee according to the National Institutes of Health (NIH) Protocol for Animal Care and Use (NIH, Bethesda, MD, USA and all efforts were made to minimize the number of animals used in the study. This study was conducted using two different potencies of NM, namely 2 and 3 mg/Kg.

*Statistical analysis*

Data were reported as mean ± SD. Statistical analysis of differences between the samples was performed using one-way ANOVA and an appropriate post-test, if necessary, using GraphPad Prism (Version 5.4 software). A 0.05 level of probability was taken as the level of significance.

**Figure 1:**
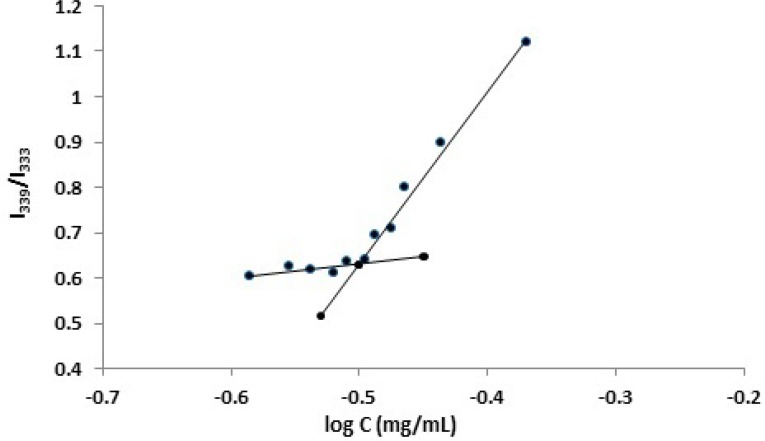
Plot of I339/I333 vs. log of P85 concentration in DI water. As depicted, the CMC value was related to the intensity ratio of the emission spectra profile

**Figure 2:**
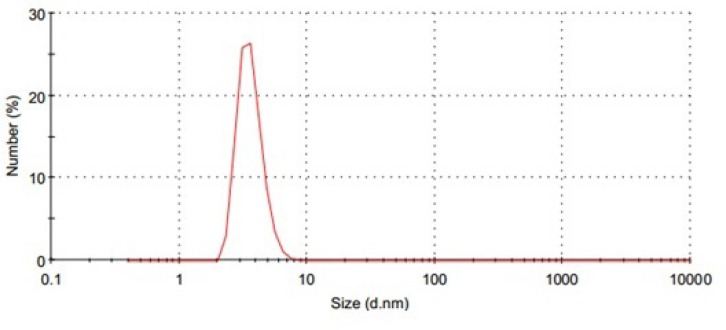
Size distribution of P85 blank micelles (10 % w/v).

**Figure 3:**
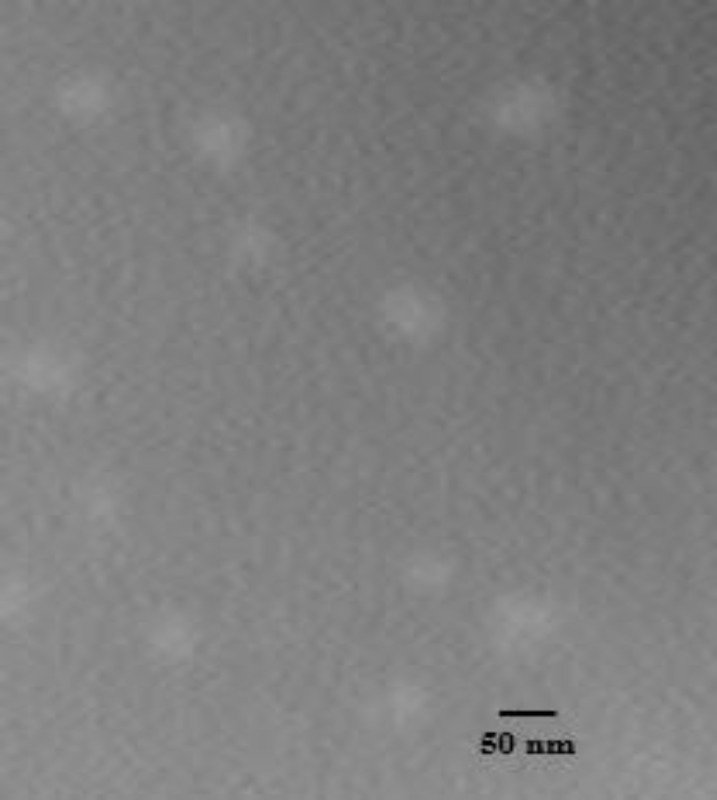
Transmission electron photomicrographs of the blank P85 polymeric micelles (F4). The scale bar represents 50 nm

**Figure 4:**
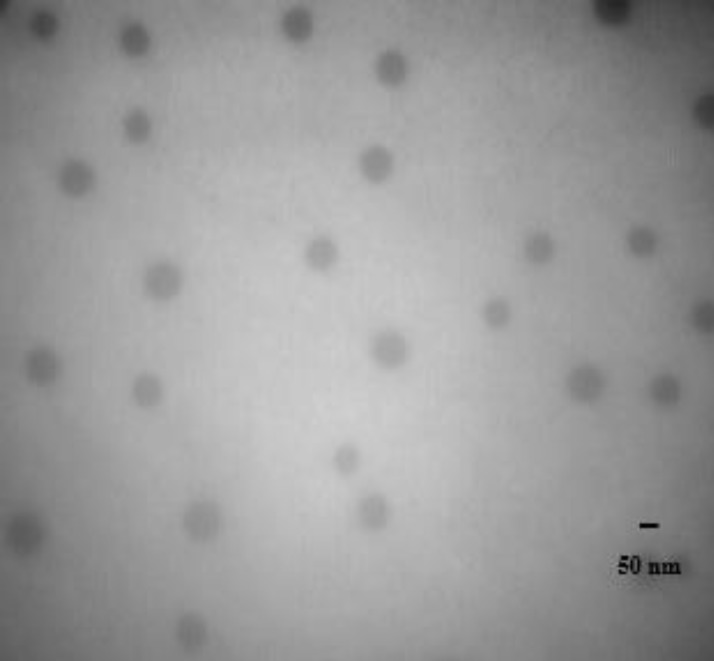
Transmission electron photomicrographs of the NM-loaded P85 polymeric micelles (F4). The scale bar represents 50 nm

**Figure 5:**
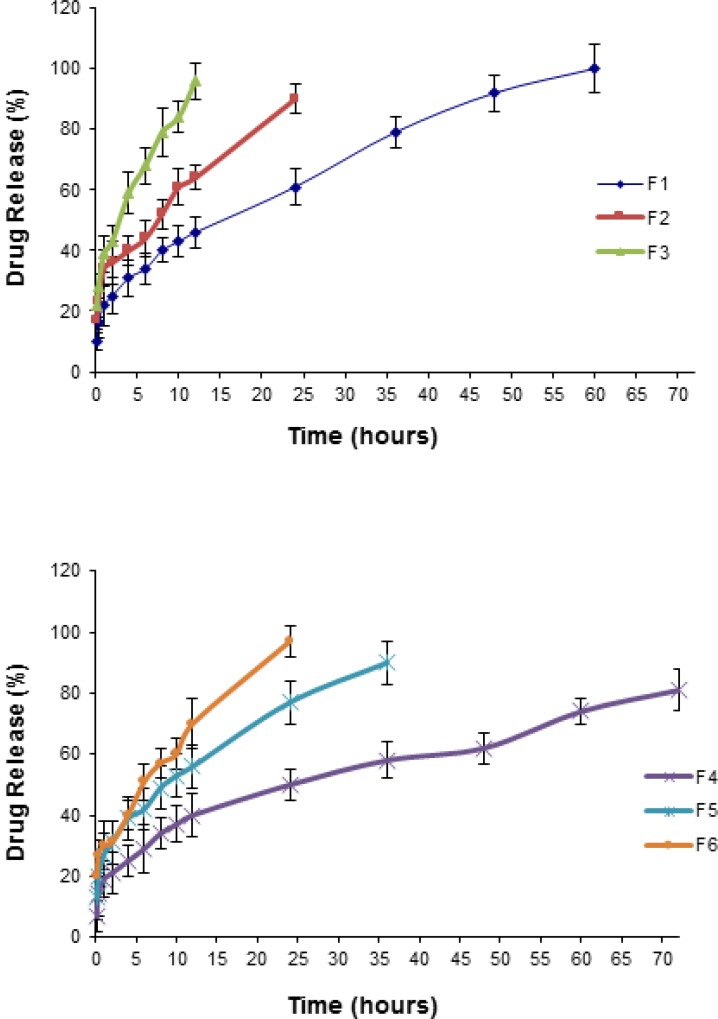
Release profiles of NM from micelles fabricated with various concentrations of Pluronics in PBS (pH 7.4) at 37 °C (Mean ± SD; n = 3

**Figure 6:**
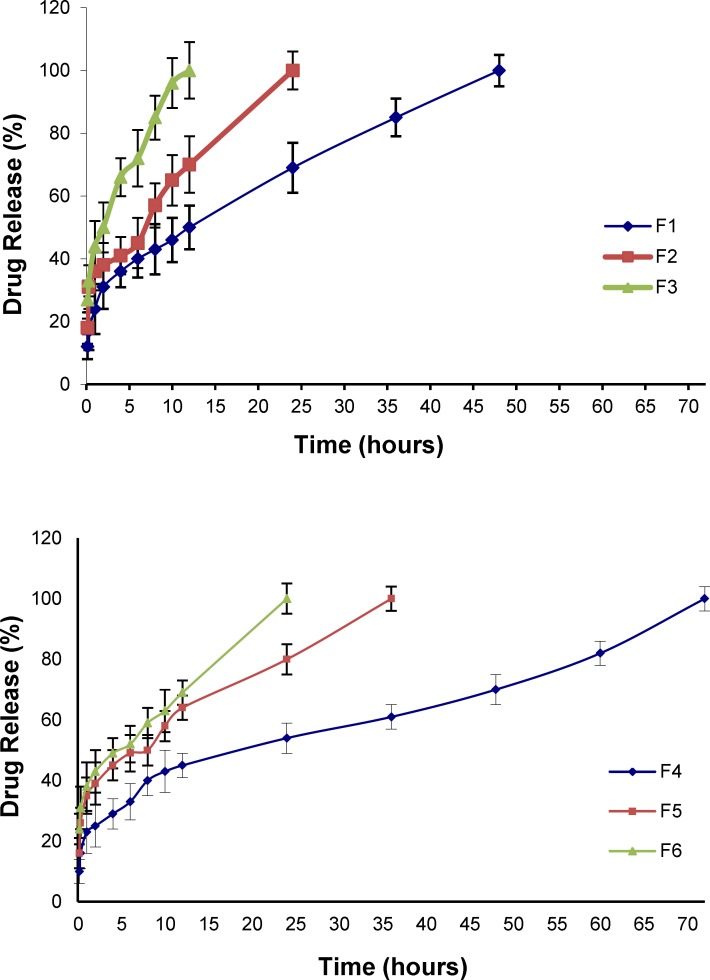
Release profiles of NM from micelles fabricated with various Pluronics in CSF at 37 °C (Mean ± SD; n = 3

**Table 1:** Features of three Pluronic block copolymers used in this study

**Copolymer**	**PEO-PPO-PEO Structure**	**MW** a **(Da)**	**HLB** b
Pluronic^® ^F68	EO_76_–PO_29_–EO_76_	8400	29
Pluronic^® ^P85	EO_26_–PO_40_–EO_26_	4600	16
Pluronic^® ^F127	EO_100_–PO_65_–EO_100_	12600	22

a The average molecular weight were provided by the manufacturer.

b Hydrophilic-lipophilic balance values of the copolymers were determined by the manufacturer.

**Table 2:** Composition of the blank polymeric solutions prepared with various Pluronics

**Formulation**	**Pluronic** ^® ^ **P85** **(% w/v)**	**Pluronic** ^® ^ **F127** **(% w/v)**	**Pluronic** ^® ^ **F68** **(% w/v)**
F_1_	5	-	-
F_2_	-	5	-
F_3_	-	-	5
F_4_	10	-	-
F_5_	-	10	-
F_6_	-	-	10

**Table 3:** Size distribution parameters of the blank polymeric micelles prepared with variou Pluronics (Mean   SD).s

**Formulae**	**Z-Average (nm)**	**Polydispersity index**	**80% Intensity**
F_1_	62.3 ± 3.1	0.20 ± 0.02	50.1 ± 3.4
F_2_	38.4 ± 2.3	0.24 ± 0.06	35.1 ± 4.8
F_3_	26.2 ± 3.6	0.81 ± 0.10	5.90 ± 2.8
F_4_	46.2 ± 2.2	0.32 ± 0.09	36.8 ± 7.5
F_5_	25.9 ± 2.5	0.26 ± 0.06	22.5 ± 4.8
F_6_	14.4 ± 2.8	0.90 ± 0.20	4.30 ± 2.1

**Table 4:** Mean particle size of NM-loaded polymeric micelles prepared by thin film hydration method (Mean   SD).

**Formulae**	**Solvent / separation method** *					
	**1**	**2**	**3**	**4**	**5**	**6**
F_1_	95.9 ± 4.2	79.5 ± 4.8	701.4 ± 17.7	122.7 ± 5.9	115.5 ± 5.8	745.8 ± 20.9
F_2_	64.7 ± 5.9	57.7 ± 1.0	656.6 ± 30.5	81.5 ± 3.8	72.7 ± 4.0	693.5 ± 17.4
F_3_	49.5 ± 5.0	39.3 ± 3.2	611.8 ± 21.8	60.6 ± 4.0	44.3 ± 4.2	641.0 ± 30.3
F_4_	77.6 ± 3.0	69.4 ± 3.7	768.7 ± 29.3	97.4 ± 3.9	93.4 ± 3.7	818.7 ± 22.7
F_5_	48.5 ± 3.9	44.2 ± 4.9	712.2 ± 12.2	63.3 ± 4.7	55.2 ± 5.9	752.1 ± 38.2
F_6_	26.9 ± 4.0	25.9 ± 5.4	672.3 ± 15.4	40.7 ± 2.5	30.9 ± 3.4	704.6 ± 11.5

2: Methanol and centrifugation

3: Methanol and filter-centrifugation

4: Acetonitrile and membrane filter

5: Acetonitrile and centrifugation

6: Acetonitrile and filter-centrifugation

* 1: Methanol and membrane filter

**Table 5:** Mean particle size of NM-loaded polymeric micelles prepared by direct dissolution method (Mean   SD).

**Formulae**	**Preparation method** *					
	**1**	**2**	**3**	**4**	**5**	**6**
F_1_	99.1 ± 6.6	127.9 ± 5.5	83.8 ± 4.9	104.1 ± 6.6	91.9 ± 7.7	93.8 ± 5.6
F_2_	70.8 ± 4.8	85.3 ± 3.7	59.3 ± 7.1	83.5 ± 4.7	69.4 ± 5.0	75.5 ± 2.8
F_3_	48.6 ± 3.1	58.1 ± 4.9	40.6 ± 2.9	57.0 ± 5.9	38.5 ± 4.9	42.2 ± 7.8
F_4_	82.5 ± 5.9	111.7 ± 7.6	79.4 ± 4.7	94.8 ± 5.5	86.0 ± 6.8	90.1 ± 4.7
F_5_	48.2 ± 4.7	68.5 ± 3.7	49.0 ± 4.8	66.2 ± 4.7	53.3 ± 5.0	53.7 ± 2.6
F_6_	24.2 ± 5.9	36.4 ± 6.7	29.9 ± 2.0	40.9 ± 3.8	26.2 ± 3.1	29.4 ± 3.8

2: Method 1 and 12 h mixing

3: Method 1 and 24 h mixing

4: method 2 and 3 h mixing

5: method 2 and 12 h mixing

6: method 2 and 24 h mixing

* 1: Method 1 and 3 h mixing

**Table 6:** The percentages of DL, EE and precipitated drug for NM-loaded polymeric micelles prepared by thin film hydration and direct dissolution methods (Mean   SD; n = 3).

**Method**	**Preparation /** **separation condition**	**EE (% )**	**DL (% )**	**Precipitated drug (% )**
TFH* /Methanol	membrane filter	24.0 - 83.9	0.08 - 0.25	9.7 - 70.1
	centrifugation	25.5 - 75.9	0.08 - 0.21	16.5 - 68.1
	filter-centrifugation	6.6 – 20.4	0.06 - 0.014	71.8 - 80.0
TFH /Acetonitrile	membrane filter	10.4 - 74.2	0.04 - 0.20	17.8 - 83.7
	centrifugation	10.4 - 69.2	0.04 - 0.16	28.9 - 80.8
	filter-centrifugation	8.1 - 14.5	0.02 - 0.05	70.1 - 83.4
DD/ Method 1	3 h mixing	7.1- 46.9	0.020 - 0.108	42.0 - 83.8
	12hr mixing	18.8 - 72.0	0.053 - 0.176	20.4 - 73.7
	24 h mixing	19.5 - 74.8	0.064 - 0.205	14.7 - 69.6
DD/ Method 2	3 h mixing	35.7 - 88.1	0.107 - 0.237	10.4 - 55.3
	12 h mixing	38.4 - 82.3	0.095 - 0.230	9.5 - 58.2
	24 h mixing	33.9 - 85.5	0.091 - 0.239	8.0 - 47.3

* Thin film hydration method

Direct dissolution method

**Table 7:** Comparison of the amount of NM released from polymeric micelles into PBS and CSF media

**Formulation**	**Time for release**	
	**in PBS**	**in CSF**
F_1_	100 % in 60 h	100 % in 48 h
F_2_	90 % in 24 h	100 % in 24 h
F_3_	96 % in 12 h	100 % in 12 h
F_4_	81 % in 72 h	100 % in 72 h
F_5_	90 % in 36 h	100 % in 36 h
F_6_	97 % in 24 h	100 % in 24 h

**Table 8:** Mean particle size of the blank micelles after storage for 3 months at room temperature (Mean   SD).

**Formulation**	**1** ^st^ ** Month (nm)**	**2** ^nd^ ** Month (nm)**	**3** ^rd^ ** Month (nm)**
F_1_	56.8 ± 7.6	78.9 ± 2.8	82.9 ± 5.8
F_2_	35.1 ± 3.1	39.6 ± 6.0	35.9 ± 3.0
F_3_	42.9 ± 2.8	102.8 ± 36.0	373.3 ± 20.3
F_4_	44.1 ± 4.5	66.8 ± 5.7	75.1 ± 4.7
F_5_	27.2 ± 3.8	32.1 ± 4.9	31.5 ± 2.9
F_6_	36.7 ± 5.9	145.0 ± 13.8	495.8 ± 25.9

**Table 9:** Entrapment efficiency of NM in Pluronic micelles after storage for 3 months at room temperature (Mean   SD

**Formulation**	**1** ^st^ ** Month (%)**	**2** ^nd^ ** Month (%)**	**3** ^rd^ ** Month (%)**
F_1_	54.5 ± 6.3	57.0 ± 4.1	52.2 ± 3.4
F_2_	42.1 ± 2.9	35.1 ± 2.6	32.9 ± 4.2
F_3_	28.4 ± 3.0	18.9 ± 3.2	10.3 ± 2.8
F_4_	83.5 ± 2.7	80.9 ± 3.5	74.0 ± 3.1
F_5_	63.2 ± 3.1	61.2 ± 2.9	58.8 ± 5.9
F_6_	35.8 ± 4.5	30.0 ± 3.9	18.7 ± 3.8

**Table 10:** Mean particle size of NM-loaded Pluronic micelles after storage for 3 months at room temperature (Mean   SD).

** Formulation**	**1** ^st^ ** Month (%)**	**2** ^nd^ ** Month (%)**	**3** ^rd^ ** Month (%)**
F_1_	96.1 ± 6.9	115.7 ± 7.2	124.6 ± 12.9
F_2_	72.0 ± 4.4	65.6 ± 5.1	74.9 ± 5.4
F_3_	64.8 ± 5.2	100.3 ± 4.9	212.0 ±20.9
F_4_	86.5 ± 3.8	104.0 ± 5.9	117.7 ± 5.8
F_5_	50.7 ± 5.0	49.5 ± 6.7	55.5 ± 4.8
F_6_	54.5 ± 5.9	142.4 ± 6.8	289.4 ± 10.6

**Table 11:** Comparison of the anticonvulsant effects of NM solution and NM-loaded P85 micelles

**Injected amount of NM**	**Ratio of dead to alive rats**	
	**NM plain solution**	**NM-loaded P85 polymeric micelles**
2 mg/kg	10:0	9:1
3 mg/kg	10:0	0:10

## Results and Discussion

*Preparation of blank micellar solutions*

As mentioned earlier, PBCs consist of polyoxyethylene (PEO) and polyoxypropylene (PPO) blocks that are arranged in a basic PEO_x_–PPO_y_–PEO_x_ structure. Depending upon the number of hydrophilic and hydrophobic units, these block copolymers are characterized by various hydrophilic-lipophilic balance (HLB) values. These molecules, therefore, display surfactant properties and are capable of self-assembling into multi-molecular aggregates (known as micelles) in aqueous solutions above their CMC. The hydrophobic PPO core is separated from the aqueous phase by the hydrated shell of PEO chains. Upon dilution below the CMC, these micelles dissociate into loose aggregates or unimers within minutes (48, 49). Generally, micelle formation is an aggregation process, due to limited aqueous solubility of PBCs, which occurs reversibly. The major determinants affecting the characteristics of Pluronic micelles are the molecular weight, length and ratio of PEO/PPO units, environmental features (such as polymer concentration, temperature, ionic strength) and the compatibility of the micelle core with the entrapped molecules (13, 22, 50, 51).

In this investigation, three types of PBC, namely P85, F127, and F68, were employed for the preparation of polymeric micelle nano-carriers in order to encapsulate NM (Table 1). The total amount of the Pluronics was kept constant at 5 and 10% w/v (Table 2). Results obtained by previous studies have confirmed that PEO/PPO ratio of the polymer affect its hydrophobic/hydrophilic characteristics, such that the lower the proportion of PEO to PPO, the higher hydrophobicity of the polymer (13). Compared to F127 (PEO/PPO ratio of 200/65 with an HLB value of 22) and F68 (PEO/PPO ratio of 152/29 and an HLB value of >24), P85 has the lowest PEO/PPO ratio (52/40) and therefore possesses the highest hydrophobicity (HLB = 16). Although it has also been shown that a lipophilic polymer has a high potential for solubilization of poorly water soluble drugs, however, the major concern for its application as a nano-carrier is the instability of the aggregated structures in aqueous media, possibly due to the formation of large lamellar structures (21, 43, 51). Lee *et al*., have demonstrated that due to the hydrophobic interactions forming a supra-macromolecular structure, the hydrophobic PPO block of Pluronic L121 was aggregated in aqueous solution and the short hydrophilic PEO block of the polymer could not provide sufficient steric hindrance to form a stable dispersion. They investigated the influence of HLB values of polymers on the stability of mixed micelles and suggested that the combination of both hydrophobic and hydrophilic copolymers at the proper ratio would increase the kinetic and thermodynamic stability of mixed nano-carriers, through tight hydrophobic interactions with hydrophobic PPO moiety and minimizing the micelle aggregation, simultaneously (52).

*Determination of CMC*

CMC, as an important characteristic for an amphiphilic copolymer, refers to the thermodynamic stability of a micellar solution. The formation of core/shell micelles with PBC can be examined by the measurement of CMC values, using a fluorescence technique. Fluorescence measurements were carried out using pyrene as a fluorescent probe to obtain the evidence of micelle formation (53-58). With an increase in the polymer concentration, the total fluorescent intensity was also increased and a shift in the fluorescence spectrum was observed. This is attributed to the affinity of pyrene for partitioning from the aqueous surrounding phase into the hydrophobic domains of the micelles once formed. Since the CMC value is related to the intensity ratio of pyrene in the excitation spectra, the ratio of I_339_/I_333_ was employed to determine the CMC of the polymers in water. Figure 1 illustrates a plot of the intensity ratio as a function of logarithm of the polymer concentration (log C). As seen, at low polymer concentrations, the ratio showed a slight change, whereas a considerable increase was observed when the polymer concentrations were above a certain limit, as a result of micelle formation. After calculation, the CMC value of F127 was determined to be as low as 0.039 mg/mL, suggesting a high stability of the polymeric micelles upon dilution in the body. Furthermore, the CMC values of P85 and F68 were measured and found to be 0.315 and 4.204 mg/mL, respectively. These findings show that the CMC values were influenced by the length of the hydrophobic moiety. In other words, the longer the hydrophobic segment, the easier the forming of micelle-like nanoparticles. These results were in a good agreement with those obtained by other workers (56, 58-63), however, it should be noted that different evaluation procedures of the CMC values of PBCs in aqueous solutions would give slightly different results.

These results generally confirmed that the Pluronic copolymers were capable of forming micelle-like nanoparticles in water. In comparison with the micelles prepared with low molecular-weight surfactants, polymeric micelles are generally more stable, exhibiting remarkably lower CMC values (58, 64-65). The lower CMC value indicates a strong tendency toward the formation of aggregates and in turn, shows high stability of micelles in solutions upon dilution (66). It should be noted that micelles are subject to extreme dilution upon intravenous injection. Therefore, if kinetically stable, slow dissociation allows polymeric micelles to retain their integrity and perhaps drug content in blood circulation above or even below CMC for some time, providing an opportunity to reach the target site before they decay to unimers (67).

*Particle size measurement of blank micelles*

In order to escape detection and destruction by the reticuloendothelial system clearance and therefore increase the remaining time in the systemic circulation, it has been reported that the size of the polymeric micelles must be less than 200 nm (68, 69). Particle size has been shown to directly affect the circulation time and biodistribution of the carriers (38, 68). In this study, the average micelle size and size distribution of empty single micelles were measured by DLS. The mean diameters of nanoparticles are listed in Table 3.

The following generalizations could be made, regarding the particle size results:

a) From Table 3, for F_1_ to F_6_, when the micelles were prepared with 5 and 10% w/v of the polymers, it could be seen that the mean diameter of blank micelles ranged from 14 to 62 nm. DLS studies revealed that, irrespective of the polymer concentration, Pluronic P85 aggregates have larger sizes than those prepared with pure Pluronic F127 or F68. 

b) An increase in the concentration of the polymer resulted in a significant decrease in the particle size. It seems that the mean size of the micelles was inversely related to the polymer content, such that the size decreases from 62 to 46 nm, 38 to 25 nm and 26 to 14 nm, for P85, F127 and F68, respectively, when 10% w/v of the polymer was used for the micelle formation.

PI is a quantitative measure of the width of the particle size distribution. PI value of zero indicates a highly mono-dispersed sample, whereas the value of 1 or more is an indication of high polydispersity. DLS investigations revealed a unimodal size distribution for all developed micelles (Figure 2.) and that, with an exception of F_3 _and F_6_, the other formulae followed a fairy mono-dispersed pattern of size distribution (PI value from 0.20 to 0.45). By comparison of the data, it could be concluded that the particle size depended upon the chemical composition of the amphiphilic PBCs, such that the particle size was decreased as the hydrophilic component in the polymer composition was increased (42, 43, 53). In this work, however, F_3_ and F_6_ systems showed a very wide particle size distribution. This could be explained by considering the tendency of PEO chain to cluster in aqueous solution, and therefore, form large and loose thermodynamically reversible aggregates (53).

*Morphological examination of blank micelles*

To characterize the morphology of blank PBC nanoparticles, TEM measurement was carried out. Representative photomicrograph of the blank P85 polymeric micelles (F_4_) is illustrated in Figure 3. As evidenced, developed micelles were found to possess nearly spherical shape of moderate uniform particle size and a smooth surface. The estimated particle size from TEM images was in good agreement with that measured by the laser scattering technique.

*Preparation and characterization of NM-loaded micellar solutions*

Polymeric micelles containing NM were prepared by thin-film hydration and direct dissolution methods. In the former method, the undissolved drug and polymer aggregates were removed by various techniques, including membrane filter, centrifugation and filtration-centrifugation. In the latter, either dissolving the polymer and drug simultaneously or individually, the precipitated NM was separated from polymeric micelle solutions by filtering through filter membranes. Particle size, PDI, % EE and % DL were then measured in the filtered, centrifuged and filter-centrifuged polymeric micelle solutions and the obtained sediments were dissolved in methanol and assayed. 

Tables 4 and 5. summarize the particle size data for polymeric micelles prepared by different methods. Regardless of the method used for the drug loading, DLS studies revealed that the loading of NM in micelles visibly affected their size and size distribution. Particle size measurements showed that the size of the drug-loaded micelles was greater than the corresponding unloaded micelles which can be correlated with the enlargement of the micellar hydrophobic core region following the drug entrapment. The following generalizations could be made, regarding the particle size results:

a) In most cases, the average sizes of the drug loaded micelles were less than 100 nm.

b) Irrespective of the polymer concentration, Pluronic P85 aggregates have larger sizes than those prepared with pure Pluronic F127 or F68. 

c) Polymeric micelles prepared with thin-layer hydration technique and then filter-centrifuged, seemed to be unstable, revealing the formation of large aggregates (ca. 445-819 nm).

d) In most cases, an increase in the concentration of the polymer from 5 to 10% w/v resulted in a significant decrease in the particle size.

e) Size analysis revealed a single peak for all developed drug-loaded micelles, and, with an exception of those samples prepared with thin-layer hydration technique and filter-centrifuged, the other formulae followed a fairy narrow pattern of size distribution (PI value from 0.11 to 0.48) (data are not shown).

f) Methanol and acetonitrile were used for loading the drug into the micelles by thin film hydration method. In contrast to methanol, acetonitrile caused bigger particles to form.

g) In the method 1 of the direct dissolution, the mean diameter of the micelles increased when the polymer-drug mixture was stirred for 12 h, whereas by using the method 2, the larger particles were achieved when the mixtures were stirred for 3 h.

Micellar core serves as a nano-reservoir for loading of water-insoluble molecules in which they can be encapsulated by chemical conjugation with the block copolymer or physical entrapment. Molecular volume of the solubilizate, its interfacial tension against water, length of the core and shell-forming blocks in the copolymer, the polymer and solubilizate concentration and the partition coefficient of the entrapped molecule between the micellar core and the surrounding aqueous medium are the most important factors affecting the extent of drug incorporation through physical means. Physical entrapment can occur mainly from hydrophobic interactions, hydrogen bond and van der Waals forces. In the NM loaded micelles, the hydrophobic effects might be the main force to entrap NM into micelle (12, 38, 67, 70-74).

NM-loaded micelles were found to possess nearly spherical shape and a smooth surface as evidenced from the TEM analysis (Figure 4.), suggesting that small-sized, homogenous micelles were formed and dispersed in the aqueous media.

As mentioned earlier, two different solvents (methanol and acetonitrile) were employed in the process of drug loading by thin film hydration method. In contrast with methanol, acetonitrile caused the formation of bigger micelles, but decreased the amount of encapsulated drug. It was also observed that the method for separating the unloaded drug, had a considerable influence on DL and EE, such that the best results were obtained when 0.45 µ filter membrane was used. In case of separating the unincorporated drug through filter-centrifugation technique, a large amount of coagulated polymer was found which could be the reason for lowering EE and DL and raising the particle size (Tables 5, 6.). It should be mentioned that an increase in the concentration of the polymer from 5 to 10% w/v resulted in a significant increase in EE, while decreasing DL, and the highest EE was obtained for P85 micelles, compared to F68 and F127 micelles (*p*< 0.05).

Results revealed that when the direct dissolution method with simultaneous addition of the drug and polymer in DI was used for the preparation of drug-loaded polymeric micelles (method 2), both EE and DL increased significantly (*p*< 0.05), whereas the mixing time showed no effect of the EE and DL. However, higher EE and DL were achieved when the drug was added to the polymer solution (method 1) and the mixture was stirred for 12 and 24 h.

Our results in this study demonstrated that, regardless of the method used for the preparation of polymeric micelles (thin film hydration or direct dissolution), the drug encapsulation capacity improved as the ratio of the hydrophobic segment to hydrophilic segment increased. Compared to F127 and F68, P85 has the lowest PEO/PPO ratio and possesses the highest hydrophobicity (HLB = 16) and therefore an increase in EE for P85 micelles could be assigned to the higher number of hydrophobic PPO units in P85.

*In-vitro release of NM from polymeric micelles in PBS*

To study the in vitro drug release pattern from the Pluronic micelles containing physically entrapped NM (2 mg NM in 10 mL micellar solution), release characteristics were monitored by dialysis method in PBS medium under sink condition Figure 5 illustrates the plots of the cumulative release percentages of NM versus time, based on the loading amount. NM release from an ethanolic solution (23.7%) containing 0.2 mg/mL NM as the control, was also investigated and found that the drug rapidly diffused out of the dialysis membrane to the dialysis medium within the first two hours. The rate of drug release from the micelles was found to be inversely correlated to the polymer concentration. As depicted from Figure 5 the released amount of the drug was dramatically decreased (*p*< 0.05) with increasing the concentration of the Pluronics from 5 to 10% (i.e., from 60 to 72 h, 24 to 36 h and 12 to 24 h for P85, F127 and F68 micelles, respectively). In case of F4, after 72 h, about 80% of the initially incorporated drug still existed in the micelles. From the release profiles, it could be found that the drug release rate was faster at the initial stage and was reduced at the later stage. Furthermore, within all the Pluronics investigated, P85 and F68 showed the lowest and highest release rate, respectively.

Our results proved that NM could freely diffuse through the dialysis membrane. In contrast with the standard solution, the polymeric micelles released the NM very slowly into the PBS medium. The differences in the drug release rate from micelles and the plain solution gives clear indication of a prolonged drug release characteristic of the micellar systems and the stability of the drug incorporation into the micelles. 

The in vitro release behavior of a lipophilic compound from a polymeric micellar system is largely affected by its inner core with hydrophobic properties (42, 75). NM could be located in both the hydrophobic interior and the hydrophilic exterior of the Pluronic micelles. The initial burst effect might be attributed to the rapid release of drugs deposited on the surface or in the micro-channels existing in micelles (42, 76). However, since the initial burst effect is dramatically less than the steady state, it could be concluded that the drug is mainly incorporated inside the micelles. On the other hand, it is expected that NM, because of its hydrophobic character, is physically entrapped in the hydrophobic core of micelles.

The release of a drug from polymeric micelles could be influenced by many factors, including polymer degradation and molecular weight, binding affinity between the polymer and the drug, the ratio of PEO/PPO units, etc. (13, 22, 42, 77). In this research, the maximum and minimum burst effects were observed for F68 and P85 formulations, respectively. Therefore, it seems that the release rate was inversely proportional to the HLB values of the PBCs. By comparing the release profiles, it could be suggested that the developed micelles could act as a solubilizing as well as a sustained release NM carrier (13). The inner hydrophobic core of the micelles could retain NM firmly, resulting in a slow drug release rate even under the sink conditions (43). An increase in the PEO/PPO ratio would enhance the distribution of more water molecules into the core of the micelles leading to the formation of more hydrophilic channels and consequently faster drug release rates (13, 21, 25, 78). Liu *et al.* suggested that the uptake speed of the release medium is dependent upon the hydrophilicity of the polymer. They believed that following the uptake, the micelles would swell, allowing the entrapped to dissolve and slowly diffuse (79).

*In vitro release of NM from micelles in artificial CSF*

The *in-vitro* drug release pattern from the Pluronic micelles containing physically entrapped NM (2 mg NM in 10 mL micellar solution) was evaluated by dialysis method in artificial CSF medium under sink condition. Figure 6. illustrates the plots of the cumulative release percentages of NM versus time, based on the loading amount. NM release from an ethanolic solution (23.7% v/v) containing 0.2 mg/mL NM as the control, was also investigated and found that the drug rapidly diffused out of the dialysis membrane to the dialysis medium within the first two hours. As can be seen, the trend in release profiles from the micelles did not change when the PBS was replaced by the artificial CSF. However, the drug release into the artificial CSF solution was found to be faster compared to the PBS medium (Table 7).

*Micelle stability*

NM-loaded and blank polymeric micelle solutions were monitored over three month storage at 25 °C for any changes in particle size, clarity and EE% after filtration of precipitated drug. In case of instability, samples were sonicated for 2 and 30 min, in order to evaluate the reversibility polymeric micelle formation. Tables 8-10 present the data of particle size and EE measurements in the blank and drug-loaded micelles.

In general:

a) Based on the results obtained, blank F2 and F5 formulations were characterized as having both small particle size and the highest degree of stability, even after 3 months storage. 

b) In case of blank F3 and F6 formulations, the micelles were found to be unstable after 2 and 3 months storage, revealing the formation of precipitates and relatively high turbidity. Sonication did not result in stabilization of the dispersions. However, the turbidity observed in these formulations after one month and F1 and F4 formulations after 3 months was disappeared once the samples were sonicated for 3 min. 

c) Sonication time was not an important factor affecting the size and stability of the dispersions.

d) Encapsulation capacity of F1, F2 and F5 micelles remained practically unchanged even after 3 months, whereas micelles prepared with the Pluronic F68 (F3 and F6) displayed a decrease in the EE under all conditions. d) NM-loaded F2 and F5 formulations were characterized as the most stable nanoparticles. 

e) The size of micelles in F3 and F6 systems showed a significant increase during the storage.

*Transport of FITC-labeled P85 micelles into the CSF*

To determine whether the P85 micelles were capable of crossing the BBB, the polymer chains were labeled with FITC (Scheme 1). In this reaction, the hydroxyl groups of the polymer chains were first oxidized to carboxylic groups and then converted to amide groups (Scheme 1). FTIR spectrum of the Pluronic P85 did not show any peak at 1700 cm^-1^. However, the characteristic peak of the oxidized polymer was seen at 1700 cm^-1^ which was then shifted to 1680 cm^-1^ after amidation. ^1^HNMR spectrum of the FITC-P85 demonstrated multiple peaks at 1.08, 3.66 and 5.21 ppm, related to hydrogen atoms of the polymer backbone. The peaks at 7-8 ppm are attributed to the aromatic hydrogen of the aryl group (Figure 7). FITC molecules are unable to pass through the BBB (9). In contrast, FITC-loaded P85 Pluronic micelles were shown to cross the BBB.

*Evaluation of anticonvulsant effect*

The anticonvulsant activities of the NM plain solution and NM-loaded P85 polymeric micelles on the induced epileptic rats are shown in Table 11. Results confirmed that the developed polymeric micelles possessed significant anticonvulsant effects compared to the control samples.

## Conclusion

We have successfully developed small sized and stable mono polymeric micelles comprising of Pluronics^®^ P85, F127 and F68, using thin film hydration and direct dissolution techniques. Characterization studies confirmed the formation of core–shell type micelles and the incorporation of NM into the micelle core. Animal studies revealed that a significant fraction of NM dose could be transported directly to the brain by passing the BBB, following intra-peritoneal injection. With low CMC value, high encapsulation efficiency, nanometer diameter, good ability to cross the BBB and controlled release behavior, it could be concluded that the Pluronic micellar formulations developed in this investigation may be considered as a promising brain delivery system for NM. However, further pharmacokinetics and biodistribution studies should be conducted to confirm the safety and efficacy of these formulations and their brain targeting potential.

## References

[1] Brighinian MW (1977). Morphology of blood-brain interfaces. Exp. Eye Res.

[2] Schlosshauer B (1993). The blood-brain barrier; morphology, molecules, and neurothelin. Bioassays.

[3] Ricci M, Blasi P, Giovagnoli S, Rossi C (2006). Delivering drugs to the central nervous system: a medicinal chemistry or a pharmaceutical technology issue? Curr. Med. Chem.

[4] Palmer AM (2010). The role of the blood–CNS barrier in CNS disorders and their treatment. Neurobiol. Dis.

[5] Pardridge WM (2003). Brain drug targeting: the future of brain drug development. Mol. Interv.

[6] Pardridge WM (2005). The blood-brain barrier: bottleneck in brain drug development. NeuroRx.

[7] Schinkel AH (1999). P-Glycoprotein, a gatekeeper in the blood-brain barrier. Adv. Drug Deliv. Rev.

[8] Palmer AM (2011). The role of the blood brain barrier in neurodegenerative disorders and their treatment. J. Alzheimers Dis.

[9] Liu L, Venkatraman SS, Yang YY, Guo K, Lu J, He B, Moochhala S, Kan L (2008). Polymeric micelles anchored with TAT for delivery of antibiotics across the blood–brain barrier. Biopolymeres (Peptide Science).

[10] Kabanov AV, Chekhonln VP, Alakhov VYu, Batrakova EV, Lebedev AS, Melk-Nubarov NS, Arzhakov SA, Levashov AV, Morozov GV, Severin ES, Kabanov VA (1989). The neuroleptic activity of haloperidol increases after its solubilization in surfactant micelles: Micelles as microcontainers for drug targeting. FEBS Lett.

[11] Löscher W and Potschka H (2005). Blood-brain barrier active efflux transporters: ATP-binding cassette gene family. NeuroRx.

[12] Kataoka K, Harada A, Nagasaki Y (2001). Block copolymer micelles for drug delivery: Design, characterization and biological significance. Adv. Drug Deliv. Rev.

[13] Wei Z, Hao J, Yuan S, Li Y, Juan W, Sha X, Fang X (2009). Paclitaxel-loaded Pluronic P123/F127 mixed polymeric micelles: Formulation, optimization and in vitro characterization. Int. J. Pharm.

[14] Danson, S, Ferry D, Alakhov V, Margison J, Kerr D, Jowle D, Brampton M, Halbert G, Ranson M (2004). Phase I dose escalation and pharmacokinetic study of pluronic polymer-bound doxorubicin (SP1049C) in patients with advanced cancer. Br. J. Cancer.

[15] Mizumura Y, Matsumura Y, Yokoyama M, Okano T, Kawaguchi T, Moriyasu F, Kakizoe T (2003). Incorporation of the anticancer agent KRN5500 into polymeric micelles diminishes the pulmonary toxicity. Jpn. J. Cancer Res.

[16] Matsumura Y, Hamaguchi T, Ura T, Muro K, Yamada Y, Shimada Y, Shirao K, Okusaka T, Ueno H, Ikeda M, Watanabe N (2004). Phase I clinical trial and pharmacokinetic evaluation of NK911, a micelle-encapsulated doxorubicin. Br. J. Cancer.

[17] Kabanov AV, Batrakova EV, Alakhov VY (2003). An essential relationship between ATP depletion and chemosensitizing activity of Pluronic block copolymers. J. Control Release.

[18] Kabanov AV, Batrakova EV, Miller DW (2003). Pluronic block copolymers as modulators of drug efflux transporter activity in the blood–brain barrier. Adv. Drug Deliv. Rev.

[19] Calvo P, Gouritin B, Chacun H, Desmaele D, D›Angelo J, Noel JP, Georgin D, Fattal E, Andreux JP and Couvreur P (2001). Long-circulating PEGylated polycyanoacrylate nanoparticles as new drug carrier for brain delivery. Pharm. Res.

[20] Torchilin VP (2001). Structure and design of polymeric surfactant-based drug delivery systems. J. Control Release.

[21] Abdelbary GA, Tadros MI (2013). Brain targeting of olanzapine via intranasal delivery of core–shell difunctional block copolymer mixed nanomicellar carriers: In-vitro characterization, ex-vivo estimation of nasal toxicity and in vivo biodistribution studies. Int. J. Pharm.

[22] Chiappetta DA, Sosnik A (2007). Poly(ethylene oxide)–poly(propylene oxide) block copolymer micelles as drug delivery agents: improved hydrosolubility, stability and bioavailability of drugs. Eur. J. Pharm. Biopharm.

[23] Alakhov AY, Kabanov AV (1998). Block copolymeric biotansport carriers as versatile vehicles for drug delivery. Expert Opin. Investig. Drugs.

[24] Kwon GS, Okano T (1999). Soluble self-assembled block copolymers for drug delivery. Pharm. Res.

[25] Zhao YZ, Sun CZ, Lu CT, Dai DD, Lv HF, Wu Y, Wan CW, Chen LJ, Lin M, Li XK (2011). Characterization and anti-tumor activity of chemical conjugation of doxorubicin in polymeric micelles (DOX-P) in-vitro. Cancer Lett.

[26] Batrakov EV, Kabanov Av (2008). Pluronic block copolymers: evolution of drug delivery concept from inert nanocarriers to biological response modifiers. J. Control Release.

[27] Szakacs G, Paterson JK, Ludwig JA, Booth-Genthe C, Gottesmen MM (2006). Targeting multidrug resistance in cancer. Nat. Rev. Drug Discov.

[28] Kabanov AV, Batrakova EV, Alakhow VY (2002). Pluronic block copolymers for overcoming drug resistance in cancer. Adv. Drug Deliv. Rev.

[29] Miller DW, Batrakova EV, Waltner TO, Alakhov VYu, Kabanov AV (1997). Interactions of pluronic block copolymers with brain microvessel endothelial cells: evidence of two potential pathways for drug absorption. Bioconjug. Chem.

[30] Langley MS, Sorkin EM (1989). Nimodipine. A review of its pharmacodynamic and pharmacokinetic properties, and therapeutic potential in cerebrovascular disease. Drugs.

[31] Soliman GM, Sharma R, Choi AO, Varshney SK, Winnik FM, Kakkar AK, Maysinger D (2010). Tailoring the efficacy of nimodipine drug delivery using nanocarriers based on A2B miktoarm star polymers. Biomaterials.

[32] Song X, Jiang Y, Ren C, Sun X, Zhang Q, Gong T, Zhang Z (2012). Nimodipine-loaded mixed micelles: formulation, compatibility, pharmacokinetics, and vascular irritability study. Int. J. Nanomed.

[33] Xiong R, Lu W, Li J, Wang P, Xu R, Chen T (2008). Preparation and characterization of intravenously injectable nimodipine nanosuspension. Int. J. Pharm.

[34] Zhang Q, Jiang X, Jiang W, Lu W, Su L, Shi Z (2004). Preparation of nimodipine-loaded microemulsion for intranasal delivery and evaluation on the targeting efficiency to the brain Int. J. Pharm.

[35] Sun Y, Rui Y, Wenliang Z, Tang X (2008). Nimodipine semi-solid capsules containing solid dispersion for improving dissolution. Int. J. Pharm.

[36] Zhang L, Liu XD, Xie L, Wang GJ (2003). P-glycoprotein restricted transport of nimodipine across blood-brain barrier. Acta Pharmacol. Sin.

[37] La SB, Okano T, Kataoka K (1996). Preparation and characterization of the micelle-forming polymeric drug indomethacin-incorporated poly(ethylene oxide)-poly(beta-benzyl L-aspartate) block copolymer micelles. J. Pharm. Sci.

[38] Zhao L, Du J, Duan Y, Zang Y, Zhang H, Yang C, Cao F, Zhai G (2012). Curcumin loaded mixed micelles composed of Pluronic P123 and F68: Preparation, optimization and in-vitro characterization. Colloids and Surf. B Biointerfaces.

[39] Bae KH, Choi SH, Park SY, Lee Y, Park TG (2006). Thermosensitive pluronic micelles stabilized by shell cross-linking with gold nanoparticles. Langmuir.

[40] Gong J, Huo M, Zhou J, Zhang Y, Peng X, Yu D, Zhang H, Li J (2009). Synthesis, characterization, drug-loading capacity and safety of novel octyl modiﬁed serum albumin micelles. Int. J. Pharm.

[41] Zhou Q, Zhang Z, Chen T, Guo X, Zhou S (2011). Preparation and characterization of thermosensitivepluronicF127-b-poly(Ɛ-caprolactone) mixed micelles. Colloids Surf. B Biointerfaces.

[42] Ge H, Hu Y, Jiang X, Cheng D, Yuan Y, Bi H, Yang C (2002). Preparation, characterization, and drug release behaviors of drug nimodipine-loaded poly(e-caprolactone)-poly(ethylene oxide)-poly(e-caprolactone) amphiphilic triblock copolymer micelles. J. Pharm. Sci.

[43] Kulthe SS, Inamdar NN, Choudhari YM, Shirolikar SM, Borde LC, Mourya VK (2011). Mixed micelle formation with hydrophobic and hydrophilic Pluronic block copolymers: Implications for controlled and targeted drug delivery. Colloids Surf. B Biointerfaces.

[44] Lu B, Zhang Q, Wang H, Wang Y, Nakayama M, Ren D (2010). Extracellular calcium controls background current and neuronal excitability via and UNC79-UNC80-NALCN cation channel complex. Neuron.

[45] Liu L, Duff K (2008). A technique for serial collection of cerebrospinal fluid from the cisterna magna in mouse. J. Vis. Exp.

[46] Sotoudegan F, Amini M, Faizi M, Aboofazeli R Development of an HPLC method for determination of nimodipine and its metabolite in rat cerebrospinal fluid. Iran. J. Pharm. Res.

[47] Faizi M, Sheikhha M, Ahangar N, Tabatabaei Ghomi H, Shafaghi B, Shafiee A, Tabatabai SA (2012). Design, synthesis and pharmacological evaluation of novel 2-[2-(2-chlorophenoxy) phenyl]-1,3,4-oxadiazole derivatives as benzodiazepine receptor agonists. Iran. J. Pharm. Res.

[48] Alexandridis P, Holzwarth JF, Hatton TA (1994). Micellization of poly (ethylene oxid)-poly (propylene oxide) -poly(ethylene oxid) triblock copolymers in aqueous solution: thermodynamics of copolymer association. Macromolecules.

[49] Alexandridis P, Lindman B (2000). Amphiphilic block copolymers. Self-Assembly and Applications.

[50] Wang YZ, Li YJ, Han LM, Sha XY, Fang XL (2007). Difunctional Pluronic copolymer micelles for paclitaxel delivery: synergistic effect of folate-mediated targeting and Pluronic-mediated overcoming multidrug resistance in tumor cell lines. Int. J. Pharm.

[51] Oh KT, Bronich TK, Kabanov AV (2004). Micellar formulations for drug delivery based on mixtures of hydrophobic and hydrophilic Pluronic R block copolymers. J. Control Release.

[52] Lee ES, Oh YT, Youn YS, Nam M, Park B, Yun J, Kim JH, Song HT, Oh KT (2011). Binary mixing of micelles using Pluronics for a nano-sized drug delivery system. Colloids Surf. BBiointerfaces.

[53] Hu Y, Jiang X, Ding Y, Zhang L, Yang C, Zhang J, Chen J, Yang Y (2003). Preparation and drug release behaviors of nimodipine-loaded poly(caprolactone)-poly(ethylene oxide)-polylactide amphiphilic copolymer nanoparticles. Biomaterials.

[54] Dong DC, Winnik MA (1984). The pyrene scale of solvent polarities. Can. J. Chem.

[55] Turro NJ, Chung CJ (1984). Photoluminescence probes for water soluble polymers Pressure and temperature effect on a polyol surfactant. Macromolecules.

[56] Astafieva I, Zhong X, Eisenberg A (1993). Critical micellization phenomena in block polyelectrolyte solution. Macromolecules.

[57] Jones MC, Leroux J-C (1999). Polymeric micelles – A new generation of colloidal drug carriers. Eur. J. Pharm. Biopharm.

[58] Torchilin VP (2001). Structure and design of polymeric surfactant-based drug delivery systems. J. Control Release.

[59] Gao Z, Eisenberg A (1993). A model of micellization for block copolymers in solutions. Macromolecules.

[60] Shin IG, Kim SY, Lee YM, Cho CS, Sung YK (1998). Methoxypoly(ethylene glycol)/e-caprolactoneamphiphilic block copolymeric micelle containing indomethacin I Preparation and characterization. J. Control Release.

[61] Lee ES, Na K, Bae YH (2003). Polymeric micelles for tumor pH and folate mediated targeting. J. Control. Release.

[62] Kabanov AV, Batrakova EV, Alakhov VY (2002). Pluronic block copolymers as novel polymer therapeutics for drug and gene delivery. J. Control Release.

[63] Oerlemans C, Bult W, Bos M, Storm G, Nijsen JF, Hennik WE (2010). Polymeric micelles in anticancer therapy: targeting, imaging and triggered release. Pharm. Res.

[64] Wang L, Zeng R, Li C, Qiao R (2009). Self-assembled polypeptide-block-poly (vinylpyrrolidone) as prospective drug-delivery systems. Colloids Surf. B Biointerfaces.

[65] Lin J, Zhu J, Chen T, Lin S, Cai C, Zhang L (2009). Drug releasing behavior of hybrid micelles containing polypeptide triblock copolymer. Biomaterials.

[66] Kedar U, Phutane P, Shidhaye S, Kadam V (2010). Advances in polymeric micelles for drug delivery and tumor targeting. Nanomedicine.

[67] Lavasanifar A, Samuel J, Kwon GS (2002). Poly(ethylene oxide)-block-poly(L-amino acid) micelles for drug delivery. Adv. Drug Deliv. Rev.

[68] Sezgin Z, Yuksel N, Baykara T (2006). Preparation and characterization of polymeric micelles for solubilization of poorly soluble anticancer drugs. Eur. J. Pharm. Biopharm.

[69] Gaucher G, Dufresne MH, Sant VP, Kang N, Maysinger D, Leroux JC (2005). Block copolymer micelles: preparation, characterization and application in drug delivery. J. Control Release.

[70] Kwon GS (1998). Diblock copolymer nanoparticles for drug delivery. Crit. Rev. Ther. Drug Carrier Syst.

[71] Allen C, Maysinger D, Eisenberg A (1999). Nano-engineering block copolymer aggregates for drug delivery. Colloids Surf B. Biointerfaces.

[72] Nagarajan R, Barry M, Ruckenstein E (1986). Unusual selectivity in solubilization by block copolymer micelles. Langmuir.

[73] Nagarajan R, Ganesh K (1996). Comparison of solubilization of hydrocarbons in (PEO-PPO) diblock versus (PEO-PPO-PEO) triblock copolymer micelles. J. Colloid Interface Sci.

[74] Hurter PN, Hatton TA (1992). Solubilization of polycyclic aromatic hydrocarbons by poly(ethylene-propylene oxide) block copolymer micelles: effects of polymer structure. Langmuir.

[75] Kim SY, Shin IG, Lee YM, Cho CS, Sung YK (1998). Methoxypoly(ethylene glycol)/e-caprolactoneamphiphilic block copolymeric micelle containing indomethacin II Micelle formation and drug release behaviors. J. Control Release.

[76] Niwa T, Takeuchi H, Hino T, Kunou N, Kawashima Y (1993). Preparation of biodegradable nanospheres of water-soluble and insoluble drugs with DL lactide/glycolide copolymer by a novel spontaneous emulsification solvent diffusion method, and the drug release behavior. J. Control Release.

[77] Pepic I, Jalsenjak N, Jalsenjak I (2004). Micellar solutions of triblock copolymer surfactants with pilocarpine. Int. J. Pharm.

[78] Kovzlov MY, Melik-Nubarov NS, Batrakova EV, Kabanov AV (2000). Relationship between Pluronic block copolymer structure, critical micellization concentration and partitioning coefficients of low molecular mass solutes. Macromolecules.

[79] Liu Z, Liu D, Wang L, Zhang J, Zhang N (2011). Docetaxel-loaded pluronic P123polymeric micelles: in-vitro and in vivo evaluation. Int. J. Mol. Sci.

